# Mechanistic Pathways and Clinical Implications of GLP-1 Receptor Agonists in Type 1 Diabetes Management

**DOI:** 10.3390/ijms25179351

**Published:** 2024-08-29

**Authors:** Charlotte Delrue, Marijn M. Speeckaert

**Affiliations:** 1 Department of Nephrology, Ghent University Hospital, 9000 Ghent, Belgium; charlotte.delrue@ugent.be; 2 Research Foundation-Flanders (FWO), 1000 Brussels, Belgium

**Keywords:** GLP-1 receptor agonists, type 1 diabetes, β-cell preservation, cardiovascular protection, immunomodulation

## Abstract

GLP-1 receptor agonists, which were initially intended to treat type 2 diabetes patients, have demonstrated promise as an adjuvant therapy for type 1 diabetes (T1D). These medications can manage T1D by improving β-cell function, reducing glucose fluctuation, and providing cardioprotective effects. Recent research suggests that boosting cell proliferation and lowering apoptosis can help maintain the bulk of β-cells. Furthermore, GLP-1 receptor agonists have potent anti-inflammatory characteristics, improving immunological control and lowering systemic inflammation, both of which are critical for reducing autoimmune damage in T1D. Beyond glucose control, these agonists have neuroprotective qualities and aid in weight management. Combining these medications with insulin could significantly change how T1D is managed. The clinical data and biological mechanisms discussed in this review support the potential use of GLP-1 receptor agonists in T1D.

## 1. Introduction

Type 1 diabetes (T1D) is an autoimmune disease that affects the pancreatic β-cells that produce insulin, resulting in insulin insufficiency and hyperglycemia. This disorder affects millions of people worldwide, and successful glucose management involves ongoing exogenous insulin therapy. However, the best glycemic control remains elusive for many T1D patients, even with improvements in insulin delivery and administration methods. While insulin therapy is still the most effective treatment option for T1D, new drugs are desperately needed to address some of its drawbacks, including hypoglycemia and weight gain. In recent years, there has been increasing interest in investigating adjunctive medicines that can work alongside insulin to enhance glycemic control and lower the burden of T1D. These drugs try to target different pathways involved in the autoimmune process and glucose metabolism [1].

In response to food consumption, intestinal L-cells secrete glucagon-like peptide-1 (GLP-1), an incretin hormone that enhances insulin secretion in a glucose-dependent manner, decreases glucagon release, slows gastric emptying, and heightens feelings of fullness. Each of these mechanisms aids in controlling blood glucose levels and weight [2]. In the 1990s, the therapeutic potential of GLP-1 was recognized, leading to the development of GLP-1 receptor agonists as a treatment for T2D [3]. In 2005, exenatide, the first GLP-1 receptor agonist, was approved by the Food and Drug Administration (FDA). Since then, several other medications have been created with different dosage schedules, ranging from weekly to twice-daily injections, including semaglutide, dulaglutide, and liraglutide [4]. Because GLP-1 receptor agonists result in excellent glucose control, promote weight loss, and give cardiovascular protection, these drugs have completely changed the way T2D is treated [5]. This class of medications may also be useful for people with T1D by enhancing β-cell function, reducing fluctuations in blood sugar, and protecting the cardiovascular system [5,6]. GLP-1 receptor agonists show promise in lowering inflammation and providing neuroprotective benefits [2]. Furthermore, compared to many other antidiabetic drugs, GLP-1 receptor agonists pose a low risk of hypoglycemia [6].

In the present review, the use of GLP-1 receptor agonists as a supplementary therapy in the management of T1D will be discussed, along with the molecular routes via which these agents may help patients with T1D.

## 2. Mechanistic Pathways of GLP-1 Receptor Agonists in Type 1 Diabetes

### 2.1. Pancreatic Effects

Unlike dipeptidyl peptidase-4 (DPP-4) inhibitors, which extend the effects of endogenous GLP-1 by preventing its breakdown, GLP-1 receptor agonists directly imitate the pharmacological characteristics of endogenous GLP-1 [7]. GLP-1 receptor agonists enhance pancreatic function and change hormone secretion, indicating potential in the treatment of both T1D and T2D. They bind to the receptors on the remaining β-cells in T1D patients, encouraging their expansion and reducing apoptosis to preserve and improve their functionality [8]. GLP-1 receptor agonists also stimulate the regeneration of pancreatic β-cells [9]. They activate transcription factors, such as musculoaponeurotic fibrosarcoma oncogene homolog A (MafA) and pancreatic and duodenal homeobox 1 (PDX-1), which promote β-cell proliferation and insulin gene expression. This increase may improve β-cell activity and insulin release in T1D individuals [7]. Moreover, these agonists can activate several intracellular signaling pathways, including the cyclic adenosine monophosphate (cAMP) pathway, which promotes cell growth and survival [10]. Activation of the extracellular signal-regulated kinases (ERK)1/2 signaling pathway is another method that GLP-1 receptor agonists use to prevent cell death. This mechanism increases β-cell survival by phosphorylating and inactivating the pro-apoptotic protein B-cell lymphoma 2 (Bcl-2)-associated agonist of cell death (Bad) [11]. Chronic exposure to high levels of free fatty acids induces endoplasmic reticulum (ER) stress, leading to β-cell apoptosis. GLP-1 receptor agonists protect against lipotoxic ER stress by upregulating ER chaperones such as binding immunoglobulin protein (BiP) and the anti-apoptotic protein JunB, which are critical for maintaining β-cell viability [12]. Another important tool by which GLP-1 receptor agonists help T1D patients is the lowering of glucagon secretion. Because T1D patients have abnormally high glucagon levels due to a lack of insulin, hepatic glucose production is stimulated, which exacerbates hyperglycemia. GLP-1 receptor agonists decrease gluconeogenesis and stabilize blood glucose levels by blocking the production of glucagon from pancreatic α-cells [3].

### 2.2. Extrapancreatic Effects

GLP-1 receptor agonists have multiple major extrapancreatic effects that are crucial for treating T1D. These advantages include altered gastrointestinal motility, enhanced satiety, and improved cardiovascular function, all of which contribute to better disease management.

GLP-1 receptor agonists decrease postprandial glucose spikes and improve food absorption by delaying stomach emptying. Modulating enteric neural circuits enhances digestive efficiency, which is beneficial in T1D therapy, where glycemic unpredictability is a common problem [13]. Furthermore, through their interactions with central nervous system pathways, GLP-1 receptor agonists enhance satiety by decreasing the appetite and calorie intake via GLP-1 receptors in the hypothalamus and other parts of the brain that regulate appetite [14]. This interaction results in either weight reduction or stabilization for T1D patients, especially those who are experiencing weight gain as a result of intense insulin therapy [3].

GLP-1 receptor agonists have positive effects on blood pressure, lipid profiles, and endothelial function, all of which are beneficial for the cardiovascular system and especially helpful for T1D patients, who are more likely to experience cardiovascular problems [2]. They improve arterial compliance and induce vasodilation by increasing the production of nitric oxide (NO) in the cells lining the blood vessels and reducing oxidative stress [7]. These agonists possess anti-inflammatory properties that aid in reducing systemic inflammation, which is associated with atherosclerosis and cardiovascular disease [8]. Additionally, they inhibit the transformation of macrophages and reduce inflammation in the cells lining the arteries, thereby reducing plaque development and arterial inflammation [15]. GLP-1 receptor agonists are also associated with a decreased incidence of cardiovascular events and a reduction in CRP levels, which is a marker of systemic inflammation [16].

Moreover, GLP-1 receptor agonists regulate the release of pro-inflammatory cytokines, such as tumor necrosis factor-alpha (TNF-α), interleukin (IL)-6, and IL-1β, in response to vascular and immune stimuli. This cytokine lowering promotes the immune system’s anti-inflammatory condition [17]. GLP-1 receptor agonists also stimulate the growth and activation of regulatory T cells (Tregs), which are critical for maintaining immunological tolerance and preventing autoimmune responses. They boost Treg activity and diminish autoimmune attacks on β-cells, making them an effective additional therapy for diabetic control [18].

## 3. GLP-1 Receptor Agonists in Glycemic Control

A number of clinical trials (Table 1) show that GLP-1 receptor agonists can significantly improve glycemic control in persons with T1D. These drugs improve insulin therapy by increasing glucose-dependent insulin synthesis, while decreasing glucagon levels. Insulin treatment and GLP-1 receptor agonists work together, lead to steady glucose levels all day long, lower the risk of weight gain and hypoglycemia, and decrease the risk of long-term complications [2,5,6,19]. Furthermore, concurrent use of GLP-1 receptor agonists allows for lower insulin dosages, improving therapy safety overall in T1D [3,7,20,21].

### 3.1. Liraglutide

#### 3.1.1. Initial Studies on Liraglutide in Type 1 Diabetes

Regardless of residual β-cell function, liraglutide (1.2 mg daily) effectively lowered insulin dosage requirements, while maintaining or enhancing glycemic control in a limited study of 29 patients with T1D [21]. This suggests that liraglutide may function as an insulin-sparing medication without impairing the regulation of blood sugar levels. In a small randomized, double-blind, placebo-controlled trial over a 12-week period, involving 40 T1D participants with an HbA1c level of at least 8% and a normal body mass index (BMI), liraglutide significantly reduced body weight and insulin requirements but did not improve glycemic control [29]. Another randomized, double-blind, crossover study involving only 14 T1D patients showed that 3 months’ treatment with liraglutide did not promote or prolong hypoglycemia in C-peptide-positive T1D patients [30].

#### 3.1.2. Large-Scale Trials: ADJUNCT ONE and ADJUNCT TWO

The ADJUNCT ONE trial examined liraglutide, in conjunction with insulin therapy, in patients with T1D. The study included 1398 people who did not receive appropriate insulin therapy. Patients were randomly allocated to receive either a placebo or liraglutide at various doses (0.6 mg, 1.2 mg, or 1.8 mg) for 52 weeks, in addition to their regular insulin prescription. Liraglutide reduced HbA1c levels considerably at all doses, with the biggest reduction occurring at 1.8 mg. Liraglutide users lost more weight than placebo users, and the effects were stronger with higher doses. Most notably, liraglutide treatment lowered total daily insulin consumption, demonstrating its ability to conserve insulin. The incidence of severe hypoglycemia in the liraglutide and placebo groups was comparable, suggesting that liraglutide did not raise the risk of hypoglycemia when used in conjunction with insulin therapy. Gastrointestinal side effects, such as nausea and vomiting, were the most common adverse events [20].

The ADJUNCT TWO trial evaluated the safety and efficacy of capped insulin titrated to liraglutide. Adult T1D patients who had not responded well to insulin alone participated in this 52-week study. Liraglutide significantly improved HbA1c levels compared to placebo across all doses, with the greatest reductions at 1.8 mg. When liraglutide was used for an extended period of time, weight reduction was substantial, especially at higher doses, and the daily insulin dosage could be reduced. When liraglutide was used in place of a placebo, the incidence of severe hypoglycemia did not rise, indicating that the medication is safe when used with less insulin. The most common side effect of liraglutide was again gastrointestinal distress, which was well tolerated [22].

In the ADJUNCT ONE and ADJUNCT TWO studies, liraglutide’s glycemic safety and effectiveness were the same for all subgroups. Residual β-cell function was the main factor impacting the action of GLP-1 receptor agonists in T1D patients [31].

#### 3.1.3. Additional Studies

In a 12-week randomized, placebo-controlled clinical trial, 72 adult patients with T1D were assessed to ascertain the safety and effectiveness of augmenting their insulin prescription with liraglutide (0.6, 1.2, and 1.8 mg daily). In addition to receiving their usual dosage of insulin, participants were randomized to either liraglutide or a placebo. The primary goal was lowering baseline HbA1c levels; additional goals included body weight, daily insulin consumption, and the frequency of hypoglycemic episodes. Adding liraglutide significantly reduced HbA1c levels and led to weight loss. Furthermore, liraglutide showed an impact that spared insulin, lowering the daily insulin dose overall without raising the risk of hypoglycemia. Consistent with the established side effect profile of GLP-1 receptor agonists, nausea and vomiting were the most frequent adverse events [32].

Another study investigated the effects of liraglutide on stomach emptying and glycemic recovery during hypoglycemia in T1D patients. This 12-week double-blind, randomized, placebo-controlled, parallel-group study involved 20 adult T1D participants, who received either a placebo or an additional 1.2 mg of liraglutide. During hypoglycemia, both groups showed comparable rates of glycemic recovery and stomach emptying; glucagon and other counterregulatory hormone levels were unaffected by liraglutide. Although the liraglutide group saw a notable rise in heart rate, the drug did not interfere with the body’s normal processes for recovering from hypoglycemia, indicating that it can be safely used as an adjuvant therapy [25].

In a third experiment, liraglutide’s effects on sugar consumption and body composition were investigated in overweight T1D patients undergoing insulin pump therapy. This 26-week randomized controlled trial included 25 overweight people with T1D. They were randomly allocated to receive routine insulin therapy, in addition to either liraglutide (1.8 mg daily) or a placebo. Waist circumference, BMI, and body weight all decreased significantly in the liraglutide group. Furthermore, participants in the liraglutide group consumed much less added sugar, demonstrating that the medicine may affect dietary habits in addition to causing weight loss [33].

#### 3.1.4. Liraglutide in Combination Therapies

In a phase 2 study, the efficacy of treating newly diagnosed T1D patients with anti-IL-21 in addition to liraglutide was investigated. Participants with T1D and residual β-cell activity, aged 18 to 45, were randomly assigned to one of four groups: liraglutide (1.8 mg daily) plus anti-IL-21, anti-IL-21 alone, or a placebo paired with standard insulin therapy. The major goal was to track changes in stimulated C-peptide concentrations over a period of 54 weeks. The combined treatment considerably reduced the reduction in C-peptide levels (10%) as compared to a placebo (39%), indicating improved function of the β-cells. While both the anti-IL-21 and liraglutide monotherapies had some efficacy, they did not outperform the placebo when administered alone. Notably, all active therapies resulted in a larger reduction in HbA1c than placebo, with no significant differences in hypoglycemia incidents between the groups. Importantly, the combined therapy had a positive safety profile, with only minor and temporary immune cell alterations reported [34].

Liraglutide has the potential to significantly improve the closed-loop insulin administration system used to treat T1D. It helps to regulate postprandial glucose levels and reduces the risk of hypoglycemia. In a randomized crossover experiment of 15 people with T1D, each participant experienced two therapy phases: a closed-loop system for glucose management, during which liraglutide (1.2 mg daily) and insulin were administered. When liraglutide was added to the closed-loop system instead of insulin alone, mean blood glucose levels dropped significantly. The average blood glucose level in the liraglutide arm was 144.6 mg/dL, lower than in the insulin-only arm (159.7 mg/dL). In addition, liraglutide improved postprandial blood glucose profiles by dramatically lowering glucose excursions after breakfast and lunch. In addition, the liraglutide arm had a considerably reduced area under the curve (AUC) for glucagon levels after lunch and breakfast. The addition of liraglutide did not raise the risk of hypoglycemic episodes [35].

Finally, a recent meta-analysis confirmed the positive effects of liraglutide on metabolic control, weight loss, and insulin dose reduction. Liraglutide induced a decrease in HbA1c (−0.09%/mg), weight (−2.2 kg/mg), and total daily insulin (−4.32 IU/mg) [36]. Other meta-analyses [37,38] showed similar results, but also a non-significant lower risk for sever hypoglycemia [37], a significant increase in heart rate [37], and a lower blood pressure [38] with the use of liraglutide.

### 3.2. Exenatide

Exenatide was tested in 14 adult patients with long-term T1D as part of a crossover trial. For six months, individuals alternated between receiving exenatide (10 μg four times a day) and not taking any medication. The results showed that exenatide, at large doses, was effective in lowering postprandial blood glucose levels. However, it increased fasting glucose concentrations, which prevented changes in overall HbA1c levels. Importantly, exenatide significantly improved insulin sensitivity beyond what was expected from weight loss alone [39].

The MAG1C trial evaluated the safety and effectiveness of short-acting exenatide administered during lunchtime to improve glycemic control in T1D patients. This double-blind, randomized, placebo-controlled trial included 106 adult T1D patients who did not respond favorably to insulin therapy alone. For 24 weeks, participants were given subcutaneous injections of either exenatide (10 µg) or a placebo before meals. Exenatide improved overall glycemic control by significantly reducing postprandial glucose excursions compared to the placebo and somewhat decreased HbA1c levels. Furthermore, weight reduction and a decrease in the daily insulin dosage were observed with exenatide medication, all without raising the risk of hypoglycemia [23]. A network meta-analysis of 23 randomized controlled trials (n = 5151 type 1 diabetes patients) showed a decrease in body weight of 5.1 kg in the insulin + exenatide group compared with the insulin group [40]. The most frequent side effects were vomiting and nausea, which is in line with the established side effect profile of GLP-1 receptor agonists [23].

A three-part, double-blind, randomized controlled trial was conducted with eight teenage volunteers with T1D to examine the potential benefits of additional exenatide therapy. The participants were divided into two groups and given either 1.25 or 2.5 μg of exenatide, and their results were compared to those who received insulin alone. To investigate the effect of exenatide on postprandial glucose excursions, the prandial insulin dose was reduced by 20% throughout the trial. Exenatide significantly reduced glucose excursions during a 300 min period compared to insulin alone, implying a significant reduction in postprandial hyperglycemia. Exenatide exhibited potential as an adjuvant therapy for teenagers by delaying stomach emptying without influencing glucagon secretion [41].

Exenatide extended release (ER) was tested on 142 T1D patients with and without detectable C-peptide levels to determine its efficacy. Depending on their ability to produce insulin, participants were divided into two groups. One group was given a placebo, while the other group received exenatide ER (2 mg once weekly) for 24 weeks. By week 12, exenatide ER had significantly decreased HbA1c levels, especially in individuals with detectable C-peptide levels. At week 24, the progress did not, however, continue. Substantial weight loss and a reduction in the total insulin dose were both outcomes of the treatment; however, the latter did not change appreciably when body weight was taken into account. Exenatide ER provided short-term glycemic improvements and weight loss, especially in those with some residual insulin production, without raising the risk of hypoglycemia [27].

In a study investigating exenatide’s impact on glycemic variability and hypoglycemia, 30 patients with T1DM were randomly divided into two groups: one received a combination of exenatide (10 µg) and insulin, while the other continued with insulin therapy alone for four weeks. Continuous glucose monitoring measured the variability of blood sugar, while hypoglycemic clamp studies examined the reactions of hormones that counter-regulate blood sugar. When compared to the insulin-only group, the exenatide-treated group showed significant reductions in body weight, BMI, total insulin dose, and glycemic excursions. In those without residual islet function, exenatide decreased the mean amplitude of glycemic excursions (MAGE) and the coefficient of variation (CV) but had no effect on the levels of glucagon and counter-regulatory hormone during hypoglycemia [42].

### 3.3. Dulaglutide

The DIAMOND-GLP-1 trial examined the effects of dulaglutide on glycemic management and insulin microsecretion in individuals with T1DM who had residual β-cell activity. This phase 2a, double-blind, randomized, placebo-controlled trial included adults with C-peptide levels above 15 pmol/L, a BMI between 16 and 30 kg/m^2^, and a diagnosis of T1DM within the past 15 years. In addition to receiving insulin, 23 patients were randomly assigned to receive either a placebo or dulaglutide (1.5 mg once weekly). The principal aim of the investigation was to evaluate if dulaglutide might augment insulin microsecretion, as gauged by the C-peptide area under the curve (AUC) in the context of a conventional mixed meal test. From baseline to 24 weeks, the dulaglutide group’s C-peptide AUC increased. Furthermore, there was no discernable difference in HbA1c levels between the placebo and dulaglutide groups. Weight reduction was 5.6 kg higher in the dulaglutide group compared to the placebo group. This can be explained by a significant reduction in daily caloric intake, particularly from consuming less fat. In addition, they experienced fewer hypoglycemic episodes with symptoms than the placebo group. The dulaglutide group did, however, have an increase in gastrointestinal side events. Overall, the study found that dulaglutide had no detectable effect on insulin secretion or glycemic control, although it did cut calorie intake and aid in weight loss in T1D patients with some β-cell function. These findings suggest that, whereas dulaglutide has no substantial impact on β-cell function in this patient population, it may be beneficial for weight management [24].

### 3.4. Albiglutide

Pozzilli et al. investigated how albiglutide and insulin therapy altered β cell activity and glycemic control in persons newly diagnosed with T1D. This phase 2 randomized, double-blind, placebo-controlled study included persons who had been diagnosed with T1D during the previous three months. Participants received either albiglutide (30 mg once weekly) or a placebo in addition to their regular insulin regimen. Over the course of 52 weeks, a mixed meal tolerance test (MMTT) was used to evaluate C-peptide levels. Albiglutide preserved C-peptide levels moderately but significantly, suggesting a protective effect on the remaining β-cell function. Furthermore, individuals in the albiglutide group experienced a significant decrease in HbA1c levels and required less daily insulin to maintain glycemic control compared to the placebo group. Although there was weight loss observed in the albiglutide group, it was not statistically significant. Crucially, compared to the placebo, the likelihood of hypoglycemia episodes did not rise with albiglutide treatment. Overall, albiglutide was well tolerated; mild to severe gastrointestinal symptoms, like nausea and diarrhea, were the most frequent side effects. So, in newly diagnosed T1D patients, albiglutide generally showed promise in maintaining beta cell function and enhancing glycemic control without raising the risk of hypoglycemia [26].

### 3.5. Semaglutide

The Real-World STEMT trial sought to evaluate the effects of once-weekly semaglutide on weight management and metabolic control in T1D patients. This six-month trial was designed to investigate semaglutide’s efficacy as an insulin adjunctive treatment. The study included 20 T1D patients, with an average age of 46.3 years and a diabetes duration of 29.6 years. Individuals who were eligible for participation were required to maintain a stable body weight and glycemic control for at least one year prior to enrolment. The trial participants lost an average of 8.5 kg after receiving weekly dosages of semaglutide (1.0 mg). HbA1c levels decreased by 0.3% on average, with 35% of individuals seeing a fall of 0.5% or greater. Furthermore, the study discovered a 13.5% reduction in total daily insulin dosage, indicating a beneficial effect on insulin requirements. These findings suggest that semaglutide, when paired with insulin, can help T1D patients maintain their weight and improve metabolic control [43].

In the recently published FLOW trial, semaglutide lowered the risk of major renal complications and cardiovascular death in individuals with T2D and chronic kidney disease (CKD) [44]. The ongoing NCT05822609 trial is testing the effects of semaglutide on sixty T1D patients with CKD. Participants will be randomized to either a placebo or semaglutide throughout a 26-week period. This study will look into whether semaglutide improves renal outcomes such as the urine albumin-to-creatinine ratio and estimated glomerular filtration rate.

### 3.6. Tirzepatide

As an adjunctive treatment for T1D, tirzepatide (5 mg once weekly), a dual glucose-dependent insulinotropic polypeptide (GIP) and GLP-1 receptor agonist, shows promise in improving glycemic control, reducing insulin dosage, and promoting weight control. The effectiveness of tirzepatide in reducing insulin requirements and enhancing glycemic control in individuals with T1D was evaluated in an observational proof-of-concept study. A considerable decline in HbA1c levels was observed, indicating better glycemic control. There was also a significant reduction in the total amount of insulin required per day. Significant weight loss was also accomplished by the individuals, which is especially beneficial for T1D patients, who commonly battle with weight gain as a result of intensive insulin therapy. Participants typically tolerated tirzepatide well in terms of safety. Consistent with the known side effects of GLP-1 receptor agonists, nausea and vomiting were the most frequently reported gastrointestinal adverse events [45].

Adults (n = 62) with established T1D who were overweight or obese and receiving tirzepatide (5 mg once weekly) in addition to their regular insulin medication participated in another proof-of-concept observational trial. Tirzepatide dramatically lowered HbA1c levels, suggesting improved glycemic control. Given the significant decrease in daily insulin requirements, it appears that tirzepatide may be an insulin-sparing medication. Participants lost a substantial amount of weight, which is advantageous for controlling T1D and its comorbidities [46]. Tirzepatide, when combined with the closed-loop Tandem Control-IQ system, can be used as an adjuvant drug to help adults with T1D improve their glycemic control and use less insulin. Tirzepatide effectively reduced total insulin demands, as evidenced by a significant decrease in 11 participants’ basal and bolus insulin doses in a single-center observational study. Participants also had more consistent glucose levels, indicating better glycemic control [28].

## 4. Potential Benefits beyond Glycemic Control

### 4.1. Cardiovascular Disease

Although GLP-1 receptor agonists are well known for their ability to control blood sugar levels during the treatment of diabetes, they also have advantages in cardiovascular protection (Table 2). These drugs significantly reduce the risk of major adverse cardiovascular events (MACEs) such as heart attack, stroke, and cardiovascular death, according to several cardiovascular outcome studies (CVOTs). Their ability to improve endothelial function by promoting vasodilation through increased NO production, reducing oxidative stress, and enhancing arterial compliance is responsible for their cardioprotective benefits. Furthermore, GLP-1 receptor agonists have anti-inflammatory and anti-atherogenic qualities. They do this by preventing the proliferation of smooth muscle cells and lowering inflammation, both of which lower the risk of atherosclerosis and, consequently, cardiovascular disease in diabetic individuals [5,47,48].

Dyslipidemia contributes to the increased risk of cardiovascular disease in patients with T1D. GLP-1 receptor agonists have demonstrated potential in raising high-density lipoprotein (HDL) cholesterol and decreasing levels of low-density lipoprotein (LDL) cholesterol and triglycerides, as well as improving lipid profiles in T1D patients. These changes result in a better lipid profile, which is an important component in lowering cardiovascular risk in this group [48,49]. GLP-1 receptor agonists have been related to reduced blood pressure, which is an important cardiovascular risk factor for people with T1D. These medications reduce systolic blood pressure, which lowers the total risk of cardiovascular events. They also encourage weight loss, which improves metabolic characteristics and lessens the strain on the heart, all of which are good for cardiovascular health [50,51]. Multiple mechanisms underlie the cardiovascular and lipid-modifying actions of GLP-1 receptor agonists. These substances protect the cardiovascular system by improving endothelial function, lowering oxidative stress, and having anti-inflammatory properties. These processes are essential in mitigating risk variables beyond glycemic management in T1DM, where the risk of cardiovascular consequences is prominent [47,52].

However, not all trials have confirmed this cardioprotective effect in T1D patients. A total of 108 adults with T1D participated in a randomized, double-blind, placebo-controlled experiment to examine the effects of short-acting exenatide on cardiovascular disease indicators in conjunction with insulin therapy. The participants had a BMI larger than 22.0 kg/m^2^, frequent daily insulin injections, and HbA1c levels from 7.5% to 10.0%. For 26 weeks, participants were randomly assigned to receive either a placebo or a 10 µg dose of exenatide three times a day for insulin therapy. The study aimed to investigate changes in body composition and cardiovascular indicators. Exenatide resulted in a decrease of 1.1 kg in lean body mass and 2.6 kg in total fat mass compared to the placebo group. It had no effect on circulating biomarkers associated with cardiovascular risk, such as CRP, TNF-α, IL-2, and -6, or oxidative stress indicators such as 8-oxo-7,8-dihydroguanosine, and 8-oxo-7,8-dihydro-2′-deoxyguanosine. Exenatide successfully decreased body weight, mostly by reducing fat mass, but it had little effect on the biomarkers associated with the risk of cardiovascular disease. Exenatide may help T1D patients manage their weight, but during the research period, it did not seem to have any effect on cardiovascular risk markers [53]. Another randomized, double-blind, placebo-controlled trial involved 100 adult patients with T1D who had a BMI of ≥25 kg/m^2^ and HbA1c levels of ≥8% and were randomly assigned to receive liraglutide at a dose of 1.8 mg daily or a placebo alongside their insulin therapy for 24 weeks. The main outcomes investigated were changes in cardiovascular risk markers such as heart rate, carotid intima–media thickness, pulse wave velocity, pulse pressure, and 24 h blood pressure. When compared to the placebo, liraglutide induced a 4.6 beats per minute (BPM) increase in the 24 h heart rate during therapy. During the day, the heart rate rose by 3.7 BPM, and at night, it rose by 7.5 BPM. Additionally, the liraglutide group’s diastolic nocturnal blood pressure increased by 4 mmHg. Even with these adjustments, liraglutide did not improve carotid intima–media thickness, pulse wave velocity, or pulse pressure as compared to the placebo. These findings suggest that while liraglutide can increase the heart rate, it does not significantly alter other cardiovascular risk factors in patients with longstanding T1D over the 24-week treatment period [54].

### 4.2. Overweight

GLP-1 receptor agonists can help people lose weight, particularly those who are obese or have T2D. These medications work by altering the central nervous system, reducing hunger and calorie intake while enhancing feelings of fullness and decreasing appetite. This causes significant weight loss or stabilization, while also improving metabolic profiles such as blood pressure and cholesterol. The long-term use of GLP-1 receptor agonists, such as semaglutide, has been demonstrated in studies to result in sustained weight loss, making them a viable therapy option for obesity [55,56,57].

Liraglutide (1.8 mg daily) was tested in the Lira Pump trial for its safety and effectiveness as an adjuvant treatment for overweight individuals with T1D who were treated with insulin pumps and had poor glycemic control. In this 26-week study, 100 T1D patients with insufficient glycemic control (HbA1c > 8%) and a BMI of >25 kg/m^2^ were enrolled. The study was double-blind, placebo-controlled, and randomized. In addition to their regular insulin prescription, patients were randomly assigned to receive either liraglutide or a placebo. An increase in HbA1c from baseline to 26 weeks was the main outcome. The secondary objectives included adverse events, hyperglycemia fluctuations, insulin dose requirements, and body weight changes. Furthermore, patient-reported outcomes, hypoglycemia episodes, and biomarkers such as postprandial glucagon levels were tracked. Liraglutide was associated with considerable reductions in body weight and insulin dosage requirements, but it did not significantly reduce HbA1c levels when compared to a placebo. Participants receiving liraglutide experienced an average weight loss of 6.8 kg and a decrease in bolus insulin doses compared to the placebo group. Moreover, the number of hypoglycemic events was reduced in the liraglutide group. The treatment also delayed gastric emptying, although there was no significant difference in postprandial plasma glucagon and GLP-1 concentrations between the groups [58].

The Lira-1 trial was designed to evaluate the safety and efficacy of liraglutide in conjunction with insulin therapy for persons with T1D who were overweight and had poor glycemic control. This 24-week research was double-blind, placebo-controlled, and randomized, with 100 patients from the Steno Diabetes Center in Denmark taking part. The subjects were given a placebo or liraglutide (1.8 mg daily) in addition to their regular dosage of insulin. The primary outcome that was measured was the variation in baseline and week 24 HbA1c values. Secondary outcomes included variations in body weight, insulin dosage, hypoglycemia episodes, and a host of other metabolic and patient-reported characteristics. The study discovered that the HbA1c levels were lowered similarly in the liraglutide and placebo groups. However, liraglutide significantly reduced the number of hypoglycemic events, insulin dosage, and body weight compared to the placebo. Liraglutide treatment was associated with an increase in heart rate and some gastrointestinal side effects such as nausea and dyspepsia [59].

Despite liraglutide’s efficacy in promoting weight loss, it did not alter the composition or function of subcutaneous adipose tissue in adults with T1D [60]. Liraglutide’s effects on overweight and obese T1D patients were further studied in a 26-week randomized controlled experiment to better understand the mechanisms underlying weight loss. A randomization was conducted among 85 T1D patients who were receiving insulin therapy and had no detectable C-peptide levels. The patients were randomly allocated to receive a daily placebo or 1.8 mg of liraglutide. The continuous glucose-monitoring (CGM) system was used to monitor blood glucose levels, and the study’s goals included evaluating changes in glycemic control, body weight, insulin dosage, and blood pressure. Compared to a placebo, liraglutide led to a considerable reduction in weight, with an average loss of 4.6 kg, mostly from fat mass loss. Additionally, there was a significant decrease in HbA1c levels, which fell by 0.41% from baseline, although the placebo-adjusted fall at 26 weeks was not statistically significant. Following liraglutide therapy, bolus and total insulin dosages decreased, as did systolic blood pressure. The time spent in the hyperglycemic zone reduced, but the time spent in the normal glycemic range increased considerably. In overweight and obese T1D patients, liraglutide significantly improves glycemic control, promotes weight loss, and lowers insulin requirements without increasing the risk of hypoglycemia [61].

During another 26-week trial, T1D patients received routine insulin medication along with either liraglutide (1.8 mg daily) or a placebo. The study assessed changes in eating habits, appetite perceptions, and body weight using meal diaries and questionnaires. When liraglutide was administered instead of a placebo, there was a significant weight loss, as well as a decrease in hunger, and fullness. Furthermore, liraglutide reduced the number of calories ingested, particularly the amount of added fats and sweets [62].

For people with T1D, liraglutide can be used in conjunction with other medications to provide metabolic benefits beyond just managing glucose levels. It has been shown to improve insulin sensitivity and promote weight loss. In a 24-week study of 100 adult T1D patients who were overweight and on insulin, liraglutide (1.8 mg daily) significantly reduced body weight and insulin requirements. However, comparing liraglutide to a placebo indicated no noticeable difference in HbA1c levels. Participants treated with liraglutide experienced a decrease in hypoglycemic events, in body weight by an average of 6.8 kg, and in bolus insulin doses. Additionally, the liraglutide group showed an increased heart rate but did not demonstrate significant changes in postprandial plasma glucagon and GLP-1 concentrations compared to placebo [63].

### 4.3. Neuroprotection

GLP-1 receptor agonists are considered promising drugs for preventing neurodegenerative diseases due to their metabolic and neuroprotective properties. These drugs can reduce neuroinflammation and provide protection against Alzheimer’s disease by inhibiting the production of pro-inflammatory cytokines in the brain. Furthermore, they activate signaling pathways that improve brain function and support the survival and growth of neurons. There is even evidence that these medications could halt the progression of neurodegenerative diseases. Several investigations have shown that GLP-1 receptor agonists improve cognitive performance in persons with moderate cognitive impairment [8,19,64].

### 4.4. Bone Metabolism

The impact of exenatide on bone metabolism in people with T1D has been studied. A parallel-group, randomized, double-blind trial saw 108 T1D patients on basal–bolus insulin therapy participating. In addition to their regular insulin prescription, participants received a preprandial subcutaneous injection of 10 μg of exenatide three times a day for a duration of 26 weeks. The forearm, hip, lumbar spine, and complete body were among the skeletal locations that were investigated. No significant variations in bone mineral density (BMD) were found between the exenatide and placebo groups. Furthermore, exenatide administration had no effect on indicators of bone production (amino-terminal propeptide of type I procollagen) or bone resorption (C-terminal telopeptides of type I collagen) compared to the placebo. It is interesting to note that, despite a significant average weight loss of 4.4 kg as compared to the placebo, exenatide had no discernible effect on bone metabolism. These results imply that, despite its effects on weight reduction, short-acting exenatide does not negatively impact bone health when used as an adjuvant to insulin therapy in T1D patients [65].

## 5. Future Directions and Conclusions

Aside from standard insulin therapy, research into GLP-1 receptor agonists may improve patient outcomes for patients with T1D. These agonists offer numerous benefits, including increased β-cell activity, cardioprotection, and anti-inflammatory and neuroprotective characteristics [7,19].

Future efforts to develop novel GLP-1 receptor agonists will focus on reducing side effects and improving delivery mechanisms to improve efficacy and patient compliance. In clinical trials, tirzepatide, a dual GIP/GLP-1 receptor agonist, has demonstrated improved glycemic control and decreased body weight, making it a promising alternative [55]. These advancements have the potential to drastically alter the therapeutic landscape by facilitating more customized and effective T1D treatment programs [3].

GLP-1 receptor agonists, in summary, offer a tremendous chance to solve the gaps in T1D management. They may lessen the burden of T1D-related problems and enhance patients’ quality of life when used in conjunction with conventional insulin therapy [66]. To validate their role and broaden their usage as a conventional supplementary therapy for T1D, more research and clinical studies are needed [31].

## Figures and Tables

**Table 1:** Summary of clinical trials of GLP-1 receptor agonists in type 1 siabetes.

GLP-1 Agonist	Participants	Duration	Primary Outcome	Results	Ref.
Liraglutide	1398 T1D patients on insulin.	52 weeks	Change in HbA1c	Significant reduction in HbA1c, weight loss, and lower insulin dose requirements compared to placebo.	[20]
Liraglutide	835 T1D patients with capped insulin	52 weeks	Change in HbA1c	Improved glycemic control with reduced insulin requirements, no increase in hypoglycemia.	[22]
Exenatide	106 T1D patients	24 weeks	Glycemic control, postprandial glucose levels	Significant reduction in postprandial glucose excursions and weight loss; modest reductions in HbA1c.	[23]
Dulaglutide	23 T1D patients with residual function	24 weeks	Insulin microsecretion, weight loss	Significant weight loss without significant changes in insulin microsecretion or HbA1c.	[24]
Semaglutide	20 adults with T1D	6 months	Weight change, metabolic control	Significant weight loss and reduction in daily insulin dose, with a slight decrease in HbA1c.	[25]
Albiglutide	68 adults newly diagnosed with T1D	52 weeks	Preservation of C-peptide levels	Modest preservation of β-cell function and improved glycemic control without increasing hypoglycemia.	[26]
Exenatide ER	142 T1D patients with/without C-peptide	24 weeks	Glycemic control, weight loss	Significant HbA1c reduction in C-peptide positive group and weight loss without increased hypoglycemia.	[27]
Tirzepatide	Adults with T1D using Tandem Control-IQ	24 weeks	Insulin requirements, glycemic control	Significant reduction in both basal and bolus insulin doses and improved glycemic control; notable weight loss observed.	[28]

Abbreviations: T1D, type 1 diabetes; HbA1c, hemoglobin A1c.

**Table 2:** Key findings of GLP-1 receptor agonist trials in type 1 diabetes.

Mechanism/Outcome	Description	Clinical Significance
β-cell function preservation	GLP-1 receptor agonists showed potential in preserving β-cell function in some trials, particularly with albiglutide and liraglutide.	Preserving β-cell function can help maintain endogenous insulin secretion, potentially reducing the need for exogenous insulin and improving long-term glycemic control.
Glycemic variability reduction	Trials indicated that GLP-1 receptor agonists reduce glycemic excursions, particularly postprandial spikes, and overall glycemic variability.	Reducing glycemic variability is crucial for minimizing complications and improving quality of life in T1D patients.
Insulin dose reduction	Several studies found a reduction in insulin requirements with the use of GLP-1 receptor agonists, without increasing the risk of hypoglycemia.	Lower insulin doses can reduce the risk of weight gain and hypoglycemia, making insulin therapy more manageable for patients.
Weight management	All GLP-1 receptor agonists demonstrated weight loss benefits, with significant reductions noted in liraglutide, semaglutide, and dulaglutide trials.	Weight loss is beneficial for T1D patients, who often struggle with weight gain due to intensive insulin therapy. This can improve metabolic outcomes and reduce cardiovascular risks.
Cardiovascular and metabolic benefits	GLP-1 receptor agonists provided additional benefits beyond glycemic control, including improved lipid profiles and reduced cardiovascular risks.	These benefits make GLP-1 receptor agonists attractive adjuncts to insulin therapy for comprehensive diabetes management.

Abbreviations: GLP-1, glucagon-like peptide-1; T1D, type 1 diabetes.
